# Identification of candidate genes for milk production traits by RNA sequencing on bovine liver at different lactation stages

**DOI:** 10.1186/s12863-020-00882-y

**Published:** 2020-07-09

**Authors:** Qian Li, Ruobing Liang, Yan Li, Yanxia Gao, Qiufeng Li, Dongxiao Sun, Jianguo Li

**Affiliations:** 1 College of Animal Science and Technology, Hebei Agricultural University, Lekai South Street, Baoding, 071001 China; 2 Hebei Animal Husbandry and Veterinary Institute, Baoding, 071000 China; 3 Department of Animal Genetics, Breeding and Reproduction, College of Animal Science and Technology, Key Laboratory of Animal Genetics and Breeding of Ministry of Agriculture, National Engineering Laboratory of Animal Breeding, China Agricultural University, No.2 Yuanmingyuan West Road, Beijing, 100193 China; 4 College of Veterinary Medicine, Hebei Agricultural University, Baoding, 071001 China

**Keywords:** RNA-sequencing, Liver, Candidate gene, Milk production traits, Dairy cattle

## Abstract

**Background:**

RNA-sequencing was performed to explore the bovine liver transcriptomes of Holstein cows to detect potential functional genes related to lactation and milk composition traits in dairy cattle. The bovine transcriptomes of the nine liver samples from three Holstein cows during dry period (50-d prepartum), early lactation (10-d postpartum), and peak of lactation (60-d postpartum) were sequenced using the Illumina HiSeq 2500 platform.

**Results:**

A total of 204, 147 and 81 differentially expressed genes (DEGs, *p* < 0.05, false discovery rate q < 0.05) were detected in early lactation vs. dry period, peak of lactation vs. dry period, and peak of lactation vs. early lactation comparison groups, respectively. Gene ontology and KEGG pathway analysis showed that these DEGs were significantly enriched in specific biological processes related to metabolic and biosynthetic and signaling pathways of PPAR, AMPK and p53 (*p* < 0.05). Ten genes were identified as promising candidates affecting milk yield, milk protein and fat traits in dairy cattle by using an integrated analysis of differential gene expression, previously reported quantitative trait loci (QTL), data from genome-wide association studies (GWAS), and biological function information. These genes were *APOC2*, *PPP1R3B*, *PKLR*, *ODC1*, *DUSP1*, *LMNA, GALE*, *ANGPTL4*, *LPIN1* and *CDKN1A*.

**Conclusion:**

This study explored the complexity of the liver transcriptome across three lactation periods in dairy cattle by performing RNA sequencing. Integrated analysis of DEGs and reported QTL and GWAS data allowed us to find ten key candidate genes influencing milk production traits.

## Background

In dairy cattle, milk yield and compositions are the most important economical traits that are typical quantitative characteristics controlled by multiple QTLs and polygenes simultaneously. Detection of key functional genes or causal variations on milk production traits could provide valuable molecular information for marker-assisted selection or genomic selection in dairy cattle thereby greatly shortening generation interval and increase the rate of genetic gain through pre-selecting young candidate bulls prior to progeny testing. In the past several decades, extensive quantitative trait locus (QTL) mapping, candidate genes analysis, and genome-wide association study (GWAS) have been implemented to identify QTLs, genes and mutations with large effects on the milk traits of dairy cows [1–4]. As of December 29, 2019, a large number of QTLs and genetic associations have been detected with 5093, 19,782, and 22,418 loci for milk yield, milk protein and milk fat, respectively (http://www.animalgenome.org/cgi-bin/QTLdb/). However, only a few studies, including *DGAT1* (Diacylglycerol O-Acyltransferase 1), *GHR* (Growth Hormone Receptor), and *ABCG2* (ATP Binding Cassette Subfamily G Member 2) gene have been confirmed as major genes for milk production traits in dairy cattle [2, 5, 6].

Beyond marker-QTL linkage analysis (LA) and/or linkage disequilibrium (LD) and GWAS, with the dramatically cost reductions and rapid development of next generation sequencing, RNA-sequencing (RNA-Seq) has become a commonly used approach to identify candidate genes for complex traits in human and domestic animals in recent years. Many bovine studies utilizing RNA-Seq have been conducted [7], including mammary tissue [8], embryos [9], leukocytes [10], different disease conditions [11], or factors with various nutrition traits [12]. Our previous RNA-Seq studies with mammary gland tissues from lactating Holstein cows with extremely high and low milk protein and fat percentages and from cows in non-lactation and peak of lactation identified 17 differentially expressed genes (DEGs) as promising candidates for milk composition traits [8, 13]. However, only a limited number of studies on transcriptional profiles in bovine liver have been reported until now [12, 14].

Actually, besides mammary gland epithelium, liver also plays critical roles in milk synthesis during lactation as the most important organ where the metabolism of gluconeogenesis, lipid, amino acids and other substances takes place in dairy cows [15–17]. Gluconeogenesis in liver is vital to meet glucose requirements for dairy cows in perinatal period. Dorland et al. (2009) presented the liver, as a crucial role in considerable metabolic adaptation, supported pregnancy and lactation through coordination and interconversion of nutrients [17], especially during the period of transition from late gestation to early lactation. A few genes in bovine liver involved in glucose and lipid metabolism had been detected via bovine gene microarrays [18]. Smith et al. (1998) reported that cholesterol content was increased in liver to meet lipoproteins synthesis and secretion, and then provided the mammary gland with cholesterol and triglycerides after parturition [19]. The mRNA expression of pyruvate carboxylase (PC) increased in medium and high liver fat concentration (LFC) groups than that in the low-LFC group, suggesting expressions of more genes related to gluconeogenesis than genes for lipid metabolism in early lactation [20]. Bu et al. (2017) compared the gene expression profiles between liver and mammary tissues during lactation in cows and found that liver expressed a larger number of metabolic genes, especially related to lipid, while mammary gland had more genes with regards to protein synthesis and secretion, proliferation/differentiation [21]. Our previous study detected some differentially expressed mRNAs, miRNAs and lncRNAs among different lactation stages in liver thereby predicted the competing endogenous RNAs (ceRNA) regulatory networks in Holstein cow [22].

In view of the important roles of liver in metabolism, the objective of this study was to search for key functional genes for milk production traits by performing RNA sequencing of liver samples from dairy cows in non-lactation and lactation. Here, we utilized liver biopsy transcriptome data from three Holstein cows during the dry period, early lactation and peak of lactation obtained using RNA-Seq and identified ten promising candidate genes for milk yield and milk compositions (protein and fat) in Holstein. Our findings could provide new insights into elucidation of the genetic basis for milk traits and potential molecular information for genomic selection in dairy cattle breeding.

## Methods

### Animals and liver tissue sample collection

Three healthy Chinese Holstein cows with similar body weight, milk yield, and milk composition were selected from 1300 Chinese Holstein cows fed in Baoding Hongda Animal Husbandry Limited Company in Hebei Province (Baoding, China) [22]. Liver biopsies were collected on 50-d before parturition (dry period), 10-d after parturition (early lactation) and 60-d after parturition (peak of lactation) from each cow as described in detail in our previous study [22]. As a result, a total of nine samples were obtained. All protocols for collection of the tissues of experimental individuals were reviewed and approved by Animal Welfare Committee of Hebei Agricultural University. All animals care and treatment in compliance with the “Principles of Laboratory Animal Care (NIH Publication No. 86-23, revised 1985)”. The cows after study were all alive and healthy and still produced milk.

### RNA sequencing, read alignment and identification of differentially expressed genes

The total RNA was isolated from each live tissue sample with TRIzol reagent (Life Technologies, CA, USA) and purified with RNase-free DNase (TIANGEN, Beijing, China). RNA integrity number (RIN) was detected with RNA Nano 6000 Assay Kit on Bioanalyzer 2100 system (Agilent Technologies, CA, USA) and samples with RIN values of higher than 7.0 were used as input material for the RNA sample preparations. Subsequently, sequencing libraries were generated using NEBNext® Ultra™ Directional RNA Library Prep Kit for Illumina® (New England Biolabs, MA, USA) and sequenced on Illumina HiSeq 2500 platform. After removing adapters, deleting reads containing poly-N and low quality reads from the raw data, we aligned the paired-end reads of the clean data to the cattle reference genome assembly UMD 3.1.80. Detailed descriptions for sequencing and assembling were shown previously [22].

Gene expression level was calculated as fragments per kilo base pair (kb) of transcript per million mapped fragments (FPKM). DEGs among the different lactation stages were detected and quantified using Cuffdiff (http://cole-trapnell-lab.github.io/cufflinks/cuffdiff/). FPKM value, fold changes (in log2 scale), *p*-values and q-values (false discovery rate: corrected p-values) of DEGs were reported in the output files from Cuffdiff, and q-value of < 0.05 was set as the threshold for significantly differential gene expression.

### Functional enrichment analysis of differentially expressed genes

Ensembl Gene IDs were uploaded to the DAVID Functional Annotation Tool (https://david.ncifcrf.gov/home.jsp) and Gene Ontology (GO) functional annotation and Kyoto Encyclopedia of Genes and Genomes (KEGG) were performed. *P*-value of 0.05 was set as the threshold for significantly enriched GO terms and pathways.

### Integrated analysis of the differentially expressed genes and previously reported QTL and GWAS data

To better identify candidate genes for milk traits, we further compared the physical positions on genome of DEGs with the previously reported QTLs that have been shown to be associated with five milk production traits, namely milk yield, protein yield, fat yield, protein percentage and fat percentage (Cattle QTLdb: http://www.animalgenome.org/cgi-bin/QTLdb/BT/index) and the significant SNPs for five milk traits identified by previous GWAS in dairy cattle [23]. Thereby, DEGs close to the QTL peak positions (less than 5 cM) and near significant SNPs (less than 5 Mb) were selected as candidate genes.

## Results

### Overview of RNA sequencing

We sequenced the cDNA libraries of nine liver tissue samples from three Holstein cows with three samples from each period, i.e. dry period (50-d prepartum), early lactation (10-d postpartum) and peak of lactation (60-d postpartum) using HiSeq 2500. In total, we acquired 780.38 million clean reads, with 86.71 million for each sample on average (range from 78.62 to 97.78 million). The sequencing quality values of Q20 and Q30 were 93.13 and 87.48% respectively (Additional file 1) indicating a high quality of sequencing data. Alignment of the sequencing reads against the bovine genome UMD3.1.80 yielded 84.49% of uniquely aligned reads across the nine samples (Additional file 2) which were used for further analyses. Pearson correlation between the three samples in each stage was higher than 0.935 that was considered as a high correlation indicating the high similarity of biological replicates (Additional file 3).

### Differentially expressed genes across three periods

With Cuffdiff software, a total of 204 (118 up-regulated and 86 down-regulated), 147 (106 up-regulated and 41 down-regulated) and 81 (57 up-regulated and 24 down-regulated) differentially expressed genes (DEGs, *p* < 0.05, false discovery rate q < 0.05), which were ranked in the top half expressed genes, were detected in the early lactation vs. dry period, peak of lactation vs. dry period, and peak of lactation vs. early lactation, respectively (Additional file 4 and Additional file 5). Details of the top 10 DEGs in the three comparisons and their full name as well as q-value and fold change were described in Table 1.

**Table 1:** Top 10 differentially expressed genes between each comparison of RNA-seq

Gene name	Gene description (early lactation vs. dry period)	FDR (q-value)	Log_2_FoldChange
*LRRC73*	leucine rich repeat containing 73	0.004690	inf
*GPX3*	glutathione peroxidase 3	0.004690	5.57
*APOA4*	apolipoprotein A4	0.004690	4.26
*HP*	haptoglobin	0.004690	4.07
*MFSD2*	major facilitator superfamily domain containing 2A	0.004690	3.67
*CDC42EP5*	CDC42 effector protein 5	0.004690	3.46
*SLC13A5*	solute carrier family 13	0.011199	3.22
*SMCT1*	solute carrier family 5	0.004690	3.22
*PAQR9*	progestin and adipoQ receptor family member IX	0.004690	3.14
*SFRP2*	secreted frizzled-related protein 2	0.008110	−3.62
Gene name	Gene description (peak of lactation vs. dry period)	FDR (q- value)	Log_2_FoldChange
*ISG15*	ISG15 ubiquitin like modifier	0.004690	5.09
*IFIT1*	interferon-induced protein with tetratricopeptide repeats 1	0.004690	4.98
*RSAD2*	radical S-adenosyl methionine domain containing 2	0.004690	4.24
*APOA4*	apolipoprotein A4	0.004690	3.64
*MX1*	MX dynamin like GTPase 1	0.004690	3.61
*GPX3*	glutathione peroxidase 3	0.004690	3.54
*MX2*	MX dynamin like GTPase 2	0.004690	3.42
*USP18*	ubiquitin specific peptidase 18	0.004690	3.39
*LOC100298356*	bone marrow stromal antigen 2	0.004690	3.33
*HERC6*	HECT and RLD domain containing E3 ubiquitin protein ligase family member 6	0.004690	3.15
Gene name	Gene description (peak of lactation vs. early lactation)	FDR (q-value)	Log_2_FoldChange
*ISG12(B)*	TLH29 protein precursor-like	0.004690	4.26
*RSAD2*	radical S-adenosyl methionine domain containing 2	0.004690	4.13
*IFIT1*	interferon-induced protein with tetratricopeptide repeats 1	0.004690	4.12
*ISG15*	ISG15 ubiquitin like modifier	0.004690	3.17
*FKBP5*	FKBP prolyl isomerase 5	0.004690	2.48
*MX1*	MX dynamin-like GTPase 1	0.017022	2.42
*RXRG*	retinoid X receptor, gamma	0.004690	−2.42
*ITGAD*	intrgrin, alpha D	0.004690	−2.48
*LYZ2*	lysozyme C-2	0.011199	−2.81
*HBB*	hemoglobin, beta	0.004690	−5.54

### Gene ontology enrichment and pathway analysis

To further know about the functional associations of the differentially expressed genes between lactation periods, we implemented gene ontology (GO) analysis with DAVID software (Additional file 6). Between early lactation and dry period, significantly enriched GO terms (*p* < 0.05) were mainly focused on metabolism-related functions, especially on carboxylic acid metabolic process, transport of lipid/ cholesterol/ sterol, regulation of cellular ketone metabolic process/ lipid biosynthetic process/ lipoprotein lipase activity, high-density lipoprotein (HDL) particle, lipoprotein particle, very low-density lipoprotein (VLDL) particle and small molecule/ carboxylic acid/ carbohydrate catabolic process. Of these, insulin-like growth factor binding and anion binding were the most significantly enriched GO molecular functions. Of note, the top 10 DEGs were involved in response to metabolic process and transport, cellular component organization localization.

Comparing the peak of lactation with dry period, the most significantly enriched GO categories (*p* < 0.05) were related to response to biotic stimulus/ other organism/ external biotic stimulus, immune effector process, HDL particle, plasma lipoprotein particle, lipoprotein particle, and molecular functions associated with double-stranded RNA binding, insulin-like growth factor binding, and growth factor binding. The top 10 DEGs were related to response to stimulus, metabolic process, biological regulation, cellular component organization or biogenesis, multi-organism process, and localization.

However, between peak of lactation and early lactation, the main significant GO categories (*p* < 0.05) were focused on triglyceride/ acylglycerol/ neutral lipid/ fatty acid/ monocarboxylic acid metabolic process, and molecular functions associated with fructose transmembrane transporter activity and double-stranded RNA binding. Among the TOP10 DEGs, cellular and metabolic processes, response to stimulus, biological regulation, cellular component organization or biogenesis, multi-organism process, and localization were enriched.

In addition, KEGG analysis significantly enriched 53, 39 and 46 pathways (Additional file 7), including PPAR and AMPK signaling pathways, cholesterol/ fatty acid metabolism and glycolysis/gluconeogenesis between early lactation vs. dry period, and PPAR, p53 and AMPK signaling pathways between peak of lactation and other two periods.

### Candidate genes identified by integrated analysis of RNA-Seq, reported QTL and GWAS data

To identify candidate functional genes affecting milk production traits, integrated analysis of the RNA-Seq data in this study and the previously reported genetic data including QTL mapping and GWASs was performed. First, we compared the physical position of each DEG with the position of known QTLs that have been shown to be associated with milk yield, milk protein and milk fat traits in dairy cattle from the Cattle QTLdb database (http://www.animalgenome.org/cgi-bin/QTLdb/). Then, each DEG was compared with the significant SNPs for milk traits in Holstein identified in a GWA study by Cole et al. [23]. As a result, ten common differentially expressed genes that were simultaneously close to the known QTLs (less than 5 cM) and significant SNPs (less than 5 Mb) were identified (Tables 2 and 3).

**Table 2:** Detailed information on the reported QTLs containing the 11 DEGs in bovine liver at three lactation stages

Gene name	Early vs. dry	Peak vs. dry	Peak vs. early	Position (bp)	Position (cM)^a^	Previously reported QTL			
						Distance to QTL peak (cM)	CI and peak location (cM)	Trait	QTL ID
*APOC2*	+			53,057,717–53,059,957	Chr18:67.9	1.9	54.713–76.57 (peak:66)	PY	2721
						2.3	54.713–76.57 (peak:65.6)	MY	1532
*ACADVL*	+			27,568,181–27,573,378	Chr19:47.1	4.0	14.1–60.4 (peak:43.1)	FY	10,443
*PPP1R3B*	–			24,316,623–24,317,477	Chr27:34.3	0.5	15.176–52.32 (peak:33.8)	FP	2740
*GALE*	+			129,706,509–129,711,871	Chr2:121.9	1.9	115.437–130 (peak:120)	FY	1515
*PKLR*	–			15,396,974–15,408,994	Chr3:25.7	0.7	22.61–27.41 (peak:25)	FY	2655
						0.7	22.61–27.41 (peak:25)	PP	2656
						1.7	6–32 (peak:27.41)	PP	3435
*ANGPTL4*	+	+		18,236,482–18,243,588	Chr7:20.6	2.8	16.75–39.33 (peak:17.8)	PP	3534
						4.7	16.75–39.33 (peak:15.9)	PP	3536
*CDKN1A*	–		+	10,551,755–10,568,782	Chr23:14.1	0.9	11.82–20.66 (peak:15)	MY	2590
						1.0	13.77–28.30 (peak:15.1)	MY	2568
*ODC1*	+	+		87,175,389–87,182,661	Chr11:95.0	0.1	92.17–97.57 (peak:94.9)	FY	2669
*LPIN1*		+	+	86,050,742–86,128,538	Chr11:93.8	1.1	92.17–97.57 (peak:94.9)	FY	2669
*DUSP1*		+		4,449,106–4,452,189	Chr20:5.5	2.7	0–31.86 (peak:8.238)	FY	2564
						4.6	0–20.165 (peak:10.08)	PY	2750
*LMNA*	+			14,695,234–14,724,663	Chr3:24.1	0.5	22.61–27.41 (peak:25)	FY	2655
						0.5	22.61–27.41 (peak:25)	PP	2656
						2.9	6–32 (peak:27.41)	PP	3435

^a^The linkage position was estimated relative to UMD3.1.80 and based on the QTL mapper v.2.019 at www.animalgenome.org/cgi-bin/QTLdb/. MY: milk yield; PY: milk protein yield; FY: milk fat yield; PP: milk protein percentage; FP: milk fat percentage

**Table 3:** Detailed information on the nearest and most significant SNPs from previous GWAS to the 22 differentially expressed genes in bovine liver at different lactation stages

Gene name	Early vs.	Peak vs.	Peak vs.	Gene position (bp)^a^	Nearest and most significant SNPs of GWAS			Traits	Raw *p* value
	dry	dry	early		Distance	Name	Position (bp)^b^		
*APOA1*	+		–	Chr15:27932198–27,934,085	1.77 Mb	BTB-00590603	29,702,877	FY,PY,FP,PP	7.05E-22 ∼ 4.71E-16
					3.59 Mb	BTB-00590405	31,527,773	FY,PY	4.12E-11 ∼ 1.22E-10
*ABCG8*	–			Chr11: 26156365–26,175,034	1.34 Mb	BTB-00470332	27,519,615	FY,PY	3.66E-09 ∼ 1.25E-08
					1.78 Mb	BFGL-NGS-115431	24,379,969	MY,FY,PY,FP,PP	3.18E-29 ∼ 1.92E-07
					2.46 Mb	BTB-01556917	23,700,685	PP	3.46E-12
					2.56 Mb	Hapmap59290-rs29022016	23,599,074	PY,PP	1.87E-22 ∼ 7.11E-08
					3.49 Mb	BFGL-NGS-116483	22,666,445	MY,FY,PY,FP,PP	1.60E-24 ∼ 1.14E-08
					3.57 Mb	ARS-BFGL-NGS-43804	22,588,525	MY,FY,PY,FP	2.85E-26 ∼ 5.15E-08
					4.03 Mb	ARS-BFGL-NGS-100459	22,128,569	FY,PY,PP	1.03E-24 ∼ 7.98E-08
*APOC2*	+			Chr18: 53057717–53,059,957	888.61 Kb	BFGL-NGS-117985	53,948,569	MY,FY,PY,FP,PP	9.83E-55 ∼ 1.71E-19
					910.9 Kb	Hapmap41540-BTA-43915	53,970,861	FY,PY,FP	3.07E-18 ∼ 2.19E-09
					2.83 Mb	Hapmap47618-BTA-43817	55,892,476	PP	1.07E-08
					4.11 Mb	ARS-BFGL-NGS-98028	57,174,711	FY,PY,FP,PP	1.40E-21 ∼ 1.04E-10
*APOA4*	+	+		Chr15: 27906598–27,919,592	1.78 Mb	BTB-00590603	29,702,877	FY,PY,FP,PP	7.05E-22 ∼ 4.71E-16
					3.61 Mb	BTB-00590405	31,527,773	FY,PY	4.12E-11 ∼ 1.22E-10
*SAA1*	+	+		Chr29:26696726–26,714,918	6.41 Kb	ARS-BFGL-NGS-24998	26,721,324	PP	2.96E-08
					4.19 Mb	UA-IFASA-8605	30,901,735	FY,FP,PP	1.50E-16 ∼ 1.50E-07
*PC*	+			Chr29: 45508279–45,611,042	2.89 Mb	ARS-BFGL-NGS-2893	48,501,273	FY,PY,FP,PP	1.98E-12 ∼ 8.87E-08
					3.28 Mb	Hapmap40456-BTA-66218	48,895,237	FY	1.35E-08
					3.75 Mb	BFGL-NGS-117323	49,357,135	MY,FY,PY,FP	4.32E-19 ∼ 3.58E-08
*PPP1R3B*	–			Chr27: 24316623–24,317,477	259.32 Kb	ARS-BFGL-NGS-43776	24,576,801	MY,FY,PY,FP,PP	6.30E-26 ∼ 1.20E-07
					4.07 Mb	BTB-01063707	28,386,241	MY,FY,PY,FP,PP	1.55E-45 ∼ 1.35E-17
*GALE*	+			Chr2:129706509–129,711,871	1.27 Mb	BTA-31250-no-rs	128,437,731	PY,FP,PP	1.26E-24 ∼ 3.25E-08
*SDS*	+			Chr17: 63302876–63,311,105	162.16 Kb	BFGL-NGS-110646	63,140,712	FY,PY,FP,PP	2.61E-14 ∼ 3.54E-08
					230.59 Kb	Hapmap52830-rs29014800	63,541,690	FP,PP	4.31E-11 ∼ 3.38E-07
					1.41 Mb	BTB-00682411	61,891,068	PP	2.65E-08
					1.76 Mb	BTB-01992588	65,071,599	MY,FY,PY	3.55E-15 ∼ 2.01E-10
					2.93 Mb	ARS-BFGL-NGS-17192	66,236,750	MY,PY	2.97E-15 ∼ 1.30E-12
					3.31 Mb	ARS-BFGL-NGS-34106	66,619,908	FY,FP,PP	4.65E-25 ∼ 3.68E-14
					3.36 Mb	Hapmap40427-BTA-41914	66,671,839	FP,PP	6.15E-19 ∼ 3.07E-11
*FBP2*	+	+		Chr8:82396095–82,438,817	1.45 Mb	BTA-14515-no-rs	83,888,935	FY,PY,PP	6.36E-12 ∼ 7.78E-09
					3.95 Mb	ARS-BFGL-NGS-20324	86,384,898	FY,PY,FP,PP	7.96E-22 ∼ 3.80E-14
*PKLR*	–			Chr3:15396974–15,408,994	1.31 Mb	UA-IFASA-8925	14,084,731	PY	3.48E-07
*SGLT1*	–			Chr17: 72690558–72,741,954	3.96 Mb	BTA-90512-no-rs	68,732,546	FP,PP	6.81E-19 ∼ 4.33E-13
*GK*	+			ChrX:118110570–118,186,827	2.14 Mb	Hapmap34068-BES2_Contig513_1168	115,968,607	MY,FY,PY,FP,PP	2.30E-27 ∼ 6.86E-09
					2.47 Mb	BTA-30585-no-rs	120,654,979	FY,FP,PP	2.02E-16 ∼ 1.36E-08
					2.50 Mb	Hapmap39109-BTA-30591	120,691,496	FP,PP	6.01E-13 ∼ 1.28E-07
					2.89 Mb	Hapmap33343-BTA-111447	121,076,442	FY,PY,FP,PP	6.03E-13 ∼ 7.03E-08
					3.41 Mb	BTA-98858-no-rs	121,600,055	FY,FP,PP	7.03E-21 ∼ 1.01E-09
					3.79 Mb	Hapmap45528-BTA-102311	114,319,576	FY,FP,PP	3.01E-18 ∼ 5.13E-09
*CYP7A1*	+	+		Chr14:26348324–26,358,692	3.76 Mb	ARS-BFGL-BAC-12159	22,587,081	MY,FY,PY,PP	4.94E-24 ∼ 6.01E-11
					4.41 Mb	ARS-BFGL-NGS-3198	21,933,950	FY,PY,FP,PP	1.21E-25 ∼ 1.45E-12
*ANGPTL4*	+	+		Chr7:18236482–18,243,588	1.84 Mb	Hapmap23865-BTA-111820	16,397,706	PP	1.51E-08
					4.60 Mb	BFGL-NGS-119066	13,632,174	FP,PP	9.49E-20 ∼ 7.48E-10
					4.63 Mb	ARS-BFGL-NGS-109534	13,608,935	FP,PP	8.42E-20 ∼ 1.94E-09
					4.65 Mb	BFGL-NGS-111315	13,584,721	FY,PY,FP,PP	4.90E-29 ∼ 7.03E-12
*IGF-1R*	–	–		Chr21:7967718–8,268,246	769.19 Kb	ARS-BFGL-NGS-10704	9,037,431	FP,PP	2.60E-13 ∼ 4.77E-08
					1.07 Mb	ARS-BFGL-NGS-77061	6,900,116	PP	9.32E-10
*CDKN1A*	–		+	Chr23:10551755–10,568,782	2.68 Mb	BFGL-NGS-115177	7,874,236	MY,FY,PY	1.83E-24 ∼ 6.49E-17
					3.28 Mb	ARS-BFGL-NGS-41214	13,846,320	MY,FY,PY,PP	2.10E-24 ∼ 3.13E-11
					4.20 Mb	ARS-BFGL-NGS-17887	14,769,068	MY,FY,PY,PP	3.76E-24 ∼ 6.86E-09
*ODC1*	+	+		Chr11:87175389–87,182,661	74.93 Kb	BTA-110370-no-rs	87,257,595	FY,PY,FP	3.10E-12 ∼ 2.68E-07
					96.35 Kb	BFGL-NGS-114578	87,279,008	FY,PY	2.85E-11 ∼ 1.82E-08
					197.94 Kb	ARS-BFGL-NGS-42014	87,380,604	FY	9.34E-09
					249.36 Kb	ARS-BFGL-NGS-17731	86,926,025	FY,PY,FP	3.31E-16 ∼ 3.55E-07
					605.73 Kb	Hapmap53648-rs29021240	86,569,656	FY,PY	1.00E-10 ∼ 2.14E-08
					641.51 Kb	ARS-BFGL-NGS-70263	87,824,167	MY,FY,PY,PP	1.16E-17 ∼ 2.25E-08
					789.42 Kb	ARS-BFGL-NGS-41670	87,972,079	FY,PP	1.41E-08 ∼ 2.53E-07
					817.29 Kb	ARS-BFGL-BAC-16207	87,999,946	FY,PY,FP,PP	4.08E-10 ∼ 3.65E-07
					2.02 Mb	Hapmap52066-rs29015690	85,153,576	FY	1.10E-07
					2.16 Mb	ARS-BFGL-NGS-28030	89,338,824	FY,PY	2.08E-12 ∼ 1.33E-07
					2.19 Mb	Hapmap31724-BTA-126967	89,371,911	FY,PY,FP	1.05E-15 ∼ 1.43E-08
					2.30 Mb	ARS-BFGL-NGS-2015	84,872,349	FY,PY,FP,PP	1.83E-18 ∼ 6.84E-05
					2.35 Mb	ARS-BFGL-NGS-104652	84,828,071	MY,FY,PY	1.05E-18 ∼ 2.22E-09
					3.54 Mb	ARS-BFGL-NGS-63978	83,637,998	FY,PY,FP,PP	2.27E-15 ∼ 1.78E-07
					3.65 Mb	Hapmap40477-BTA-105881	83,526,455	FY,PY,FP,PP	1.80E-24 ∼ 2.41E-11
					4.19 Mb	Hapmap56387-rs29014077	82,982,779	FY	1.25E-07
					4.25 Mb	ARS-BFGL-NGS-93601	82,920,732	FY,PY,FP,PP	1.88E-21 ∼ 2.96E-13
					4.53 Mb	Hapmap46768-BTA-117394	82,643,114	MY,FY,PY	1.78E-13 ∼ 1.73E-07
*GADD45B*		+	+	Chr7:22411968–22,414,079	2.24 Mb	Hapmap49309-BTA-78604	24,655,689	PP	4.34E-14
*LPIN1*		+	+	Chr11:86050742–86,128,538	2.38 Kb	ARS-BFGL-NGS-14236	86,048,363	FY,PY	1.29E-12 ∼ 4.60E-08
					441.12 Kb	Hapmap53648-rs29021240	86,569,656	FY,PY	1.00E-10 ∼ 2.14E-08
					797.49 Kb	ARS-BFGL-NGS-17731	86,926,025	FY,PY,FP	3.31E-16 ∼ 3.55E-07
					897.17 Kb	Hapmap52066-rs29015690	85,153,576	FY	1.10E-07
					1.13 Mb	BTA-110370-no-rs	87,257,595	FY,PY,FP	3.10E-12 ∼ 2.68E-07
					1.15 Mb	BFGL-NGS-114578	87,279,008	FY,PY	2.85E-11 ∼ 1.82E-08
					1.18 Mb	ARS-BFGL-NGS-2015	84,872,349	FY,PY,FP,PP	1.83E-18 ∼ 6.84E-05
					1.22 Mb	ARS-BFGL-NGS-104652	84,828,071	MY,FY,PY	1.05E-18 ∼ 2.22E-09
					1.25 Mb	ARS-BFGL-NGS-42014	87,380,604	FY	9.34E-09
					1.70 Mb	ARS-BFGL-NGS-70263	87,824,167	MY,FY,PY,PP	1.16E-17 ∼ 2.25E-08
					1.84 Mb	ARS-BFGL-NGS-41670	87,972,079	FY,PP	1.41E-08 ∼ 2.53E-07
					1.87 Mb	ARS-BFGL-BAC-16207	87,999,946	FY,PY,FP,PP	4.08E-10 ∼ 3.65E-07
					2.41 Mb	ARS-BFGL-NGS-63978	83,637,998	FY,PY,FP,PP	2.27E-15 ∼ 1.78E-07
					2.52 Mb	Hapmap40477-BTA-105881	83,526,455	FY,PY,FP,PP	1.80E-24 ∼ 2.41E-11
					3.07 Mb	Hapmap56387-rs29014077	82,982,779	FY	1.25E-07
					3.13 Mb	ARS-BFGL-NGS-93601	82,920,732	FY,PY,FP,PP	1.88E-21 ∼ 2.96E-13
					3.21 Mb	ARS-BFGL-NGS-28030	89,338,824	FY,PY	2.08E-12 ∼ 1.33E-07
					3.24 Mb	Hapmap31724-BTA-126967	89,371,911	FY,PY,FP	1.05E-15 ∼ 1.43E-08
					3.41 Mb	Hapmap46768-BTA-117394	82,643,114	MY,FY,PY	1.78E-13 ∼ 1.73E-07
					4.21 Mb	ARS-BFGL-NGS-105586	81,845,043	FY,PY,FP	2.47E-15 ∼ 3.00E-08
					4.23 Mb	ARS-BFGL-NGS-52709	81,819,453	FY,PY,FP,PP	3.01E-25 ∼ 1.28E-08
*DUSP1*		+		Chr20:4449106–4,452,189	44.19 Kb	ARS-BFGL-NGS-48030	4,496,376	PP	2.07E-08
					53.45 Kb	Hapmap54098-rs29010434	4,395,656	FY,PY,PP	1.35E-12 ∼ 1.66E-08
					3.32 Mb	Hapmap49207-BTA-51446	7,770,495	MY,FY,PY,FP,PP	5.02E-27 ∼ 9.06E-11
					3.50 Mb	Hapmap36217-SCAFFOLD290026_21689	7,954,374	FY,PY	7.19E-09 ∼ 7.36E-09
					3.74 Mb	ARS-BFGL-NGS-27058	712,232	FY,PY,FP	1.11E-10 ∼ 3.92E-07
*LMNA*	+			Chr3:14695234–14,724,663	0.61 Mb	UA-IFASA-8925	14,084,731	PY	3.48E-07

^a,b^Means the position on the bovine genome sequence of UMD3.1.80

*MY* milk yield; *FY* milk fat yield; *PY* milk protein yield; *FP* milk fat percentage; *PP* milk protein percentage**.**

Thus, through combination of DEGs, QTL and GWAS data, and biological functions, such ten genes were suggested as promising candidates for milk production traits, i.e. *APOC2* (*Apolipoprotein C2*), *PPP1R3B* (*Protein phosphatase 1 regulatory subunit 3B*), *PKLR* (*Pyruvate kinase*), *ODC1* (Ornithine decarboxylase 1), *DUSP1* (*Dual specificity phosphatase 1*) and *LMNA* (Lamin), *GALE* (*UDP-galactose-4-epimerase*), *ANGPTL4* (*Angiopoietin like 4*), *LPIN1* (*Lipin 1*), and *CDKN1A* (*Cyclin dependent kinase inhibitor 1A*).

## Discussion

In this study, we obtained the liver transcriptome profiles in different lactation periods of Holstein cows using high-throughput RNA sequencing, and 432 differentially expressed genes (DEGs), which were ranked in the top half expressed genes, were detected among the dry period (−50 day), early lactation (+ 10 day), and peak of lactation (+ 60 days). The DEGs expressed in the bottom half level were eliminated to ensure the detection power. Rapaport et al. (2013) investigated the relationship between detection power of DEGs and sequence depth and number of replicates, and demonstrated that with most methods, over 90% of differently expressed genes at the top expression levels are detected with little as 2 replicates and 5% of the reads [24]. Trapnell et al. (2013) also reported that the detection rate of DEGs was similar for three or more replicates using Cuffdiff [25]. Of noted, the more biological replicates are taken, more detection power are improved.

Through functional enrichment, we found that the differentially expressed genes (DEGs) between milking and non-lactation status (early and peak of lactation vs before calving) mainly participated in metabolisms of lipid, fatty acid, protein, carbohydrate and energy, and involved in MAPK, p53 and PPAR signaling pathways. Especially, lipid metabolism was significantly enriched such as fat digestion and absorption, fatty acid metabolism, bile secretion, endocytosis, biosynthesis of unsaturated fatty acids, endocytosis, fatty acid metabolism, AMPK, and PPAR signaling pathways. This is mostly likely due to milk lipids, proteins, lactose, saturated and unsaturated fatty acids that need to be synthesized during lactation. These results also suggested that metabolism and synthesis in liver provided nutrient substance support for the milking in mammary gland probably through the milking vein in dairy cows. Previous studies also revealed that the contents of triacylglycerol [26] and blood nonesterified fatty acids (NEFA) [27] were increased greatly during the perinatal period.

Based on the integrated analysis of DEGs, QTLs, GWAS data and biological functions, a total of ten promising candidate genes were found. Among them, four genes like *GALE*, *ANGPTL4*, *LPIN1*, and *CDKN1A* have been reported to play roles in milk production [28]. Six novel candidate genes identified in this study included *APOC2, PPP1R3B*, *PKLR*, *ODC1*, *DUSP1*, and *LMNA*.

APOC2, encoded by the *APOC2* gene, is the component of chylomicrons (CM), very low density lipoprotein (VLDLs), low density lipoprotein (LDLs) and high density lipoprotein (HDLs) in plasma. It plays an important role in lipoprotein metabolism as an activator of lipoprotein lipase. The deficiency of *APOC2* could cause high circulating levels of triglycerides (TGs), while overexpression of *APOC2* could inhibit lipoprotein lipase activity [29, 30]. In addition, *APOC2* was close to two known QTLs for milk protein yield and milk yield with distances of 1.9 to 2.3 cM and also near four significant SNPs for milk fat and protein traits detected by Cole et al.(2011) [23].

*PPP1R3B* and *PKLR* are related to carbohydrate metabolism. *PPP1R3B* activates glycogen synthesis and limits glycogen breakdown in the liver and skeletal muscle [31]. It was not only close to one reported QTL for milk fat percentage with distance of 0.5 cM, also near two significant SNPs for milk fat and protein identified by GWAS [23]. *PKLR* plays a key role in hepatic glycolysis [32]. It was close to three known QTLs (0.7 to 1.7 cM) for milk fat yield, milk protein percentage and milk fat percentage and also near one significant SNP for milk protein yield [23].

The *ODC1* gene encodes ODC1 that is a key enzyme of polyamine biosynthesis and a main regulator of protein synthesis and lactogenesis. The transcription of *ODC1* is stimulated by high nutritional levels and is elevated during periods of rapid mammary growth and differentiation [33]. *ODC1* was very close to two known QTLs for milk fat yield with 0.1 cM and near 18 significant SNPs for milk fat and protein with distances 74Kb ~ 4.53 Mb [23].

*DUSP1* is involved in the epithelial-to-mesenchymal transition, regulation of breast cancer stem cells (CSCs) and signal transduction [34]. *DUSP1* was not only close to the two known QTLs for milk fat yield and milk protein yield (2.7 to 4.6 cM) but also near five significant SNPs for milk fat and protein [23]. *LMNA* plays an important role in nuclear assembly, chromatin organization, nuclear membrane formation and telomere dynamics. The mutations in *LMNA* caused an imbalance between lipid oxidation and oxidative glucose metabolism in skeletal muscle metabolism [35]. It was near to the peak positions of three QTLs (0.5 to 2.9 cM) for milk fat yield, milk protein percentage and milk fat percentage and near one significant SNP for milk protein yield [23].

## Conclusions

This study detected significantly expressed genes (DEGs) in liver among three lactation periods (dry period, early and peak of lactation) by performing RNA sequencing in Holstein cows. Integrated analysis of DEGs, previously reported QTL and GWAS data, and biological functions of genes identified 10 promising candidate genes for milk production traits, including *APOC2*, *PPP1R3B*, *PKLR*, *ODC1*, *DUSP1*, *LMNA*, *GALE*, *ANGPTL4*, *LPIN1* and *CDKN1A*. Our findings provided a solid basis for further in-depth studies on how these genes regulate milk synthesis and molecular information for genomic selection in dairy cattle.

## Supplementary information

**Additional file 1.** The basic statistics for RNA-seq reads generated from liver tissues of three cows at different lactation stages.

**Additional file 2.** Summary of sequence read alignments to the reference genome.

**Additional file 3.** Correlation analysis of the reads.

**Additional file 4.** Volcano plot displaying differential expressed genes in bovine liver at different lactation stages.

**Additional file 5.** List of differentially expressed genes in bovine liver between early lactation and dry period/peak of lactation and dry period/peak of lactation and early lactation.

**Additional file 6.** List of significant GO terms assignment for DEGs in bovine liver between early lactation and dry period/peak of lactation and dry period/peak of lactation and early lactation.

**Additional file 7.** List of significant KEGG pathway categories for DEGs in bovine liver between early lactation and dry period/peak of lactation and dry period/peak of lactation and early lactation.

## Data Availability

All relevant data are available within the article and its additional files. The RNA sequencing datasets generated during current study are not publicly available because another program is under way, but are available from the corresponding author on reasonable request.

## Abbreviations

QTL: Quantitative trait loci
GWAS: Genome-wide association study
RNA-seq: RNA sequencing
LA: Linkage analysis
LD: Linkage disequilibrium
DEGs: Differentially expressed genes
EGs: Expressed genes
NEFA: Nonesterified fatty acids
CM: Chylomicrons
VLDL: Very low density lipoprotein
LDL: Low density lipoprotein
HDL: High density lipoprotein
