# Supplementary material for: Identification of candidate genes for milk production traits by RNA sequencing on bovine liver at different lactation stages
Source: BMC Genet. 2020 Jul 9;21:72. doi: 10.1186/s12863-020-00882-y (PMC7346489; doi:10.1186/s12863-020-00882-y)
Supplement: Supplementary file 1 — Additional file 1. The basic statistics for RNA-seq reads generated from liver tissues of three cows at different lactation stages. [file 12863_2020_882_MOESM1_ESM.docx]

Additional file **1 The basic statistics for RNA-seq reads generated from liver tissues of 3 cows at different lactation stages.**

| Sample name | Raw reads | Clean reads | Clean bases | Error rate (%) | Q20 (%) | Q30 (%) | GC content (%) |
| --- | --- | --- | --- | --- | --- | --- | --- |
| A_1 | 86,193,692 | 83,667,860 | 10.46G | 0.04 | 92.50 | 86.55 | 47.97 |
| A_2 | 99,986,546 | 97,780,170 | 12.22G | 0.04 | 93.24 | 87.69 | 49.57 |
| A_3 | 88,265,716 | 85,888,072 | 10.74G | 0.03 | 93.47 | 88.06 | 48.72 |
| B_1 | 89,354,306 | 87,275,100 | 10.91G | 0.03 | 93.55 | 88.19 | 48.26 |
| B_2 | 94,662,560 | 92,522,210 | 11.57G | 0.04 | 93.43 | 88.01 | 48.98 |
| B_3 | 89,655,958 | 86,817,366 | 10.85G | 0.03 | 93.56 | 88.20 | 48.59 |
| C_1 | 86,962,770 | 84,045,138 | 10.51G | 0.04 | 92.59 | 86.46 | 50.39 |
| C_2 | 85,912,968 | 83,760,436 | 10.47G | 0.04 | 93.03 | 87.26 | 48.26 |
| C_3 | 80,470,996 | 78,622,660 | 9.83G | 0.04 | 92.80 | 86.89 | 48.68 |

Q20: the proportion of bases with a phred base quality score greater than 20; i.e., the proportion of read bases whose error rate is less than 1%.

Q30: the propo**rtion of bases with a** phred base quality score greater than 30; i.e., the proportion of read bases whose error rate is less than 0.1%.A, B, or C represents the different individual; 1, 2 or 3 represents dry period, early lactation, or peak of lactation.
