# Supplementary material for: Identification of candidate genes for milk production traits by RNA sequencing on bovine liver at different lactation stages
Source: BMC Genet. 2020 Jul 9;21:72. doi: 10.1186/s12863-020-00882-y (PMC7346489; doi:10.1186/s12863-020-00882-y)
Supplement: Supplementary file 2 — Additional file 2. Summary of sequence read alignments to the reference genome. [file 12863_2020_882_MOESM2_ESM.docx]

Additional file **2 Summary of sequence read alignments to the reference genome.**

| Sample Name | A_1 | A_2 | A_3 | B_1 | B_2 | B_3 | C_1 | C_2 | C_3 |
| --- | --- | --- | --- | --- | --- | --- | --- | --- | --- |
| Total reads | 83667860 | 97780170 | 85888072 | 87275100 | 92522210 | 86817366 | 84045138 | 83760436 | 78622660 |
| Total mapped | 75554821 | 88869063 | 78151929 | 79682379 | 84320339 | 78422070 | 75520246 | 76126718 | 71188535 |
|  | (90.3%) | (90.89%) | (90.99%) | (91.3%) | (91.14%) | (90.33%) | (89.86%) | (90.89%) | (90.54%) |
| Multiple mapped | 4163846 | 6420730 | 5821862 | 4612232 | 5648087 | 5796739 | 5374110 | 5377273 | 5212475 |
|  | (4.98%) | (6.57%) | (6.78%) | (5.28%) | (6.1%) | (6.68%) | (6.39%) | (6.42%) | (6.63%) |
| Uniquely mapped | 71390975 | 82448333 | 72330067 | 75070147 | 78672252 | 72625331 | 70146136 | 70749445 | 65976060 |
|  | (85.33%) | (84.32%) | (84.21%) | (86.02%) | (85.03%) | (83.65%) | (83.46%) | (84.47%) | (83.91%) |
| Read-1 | 35955123 | 41510798 | 36328342 | 37715830 | 39554238 | 36528630 | 35246347 | 35756222 | 33335431 |
|  | (42.97%) | (42.45%) | (42.3%) | (43.21%) | (42.75%) | (42.08%) | (41.94%) | (42.69%) | (42.4%) |
| Read-2 | 35435852 | 40937535 | 36001725 | 37354317 | 39118014 | 36096701 | 34899789 | 34993223 | 32640629 |
|  | (42.35%) | (41.87%) | (41.92%) | (42.8%) | (42.28%) | (41.58%) | (41.53%) | (41.78%) | (41.52%) |
| Reads map to ‘+’ | 35665274 | 41190793 | 36131035 | 37503319 | 39278623 | 36266513 | 35016224 | 35305791 | 32919263 |
|  | (42.63%) | (42.13%) | (42.07%) | (42.97%) | (42.45%) | (41.77%) | (41.66%) | (42.15%) | (41.87%) |
| Reads map to ‘-’ | 35725701 | 41257540 | 36199032 | 37566828 | 39393629 | 36358818 | 35129912 | 35443654 | 33056797 |
|  | (42.7%) | (42.19%) | (42.15%) | (43.04%) | (42.58%) | (41.88%) | (41.8%) | (42.32%) | (42.04%) |
| Non-splice reads | 48705800 | 50171385 | 42996516 | 43995311 | 48413709 | 45595764 | 42167219 | 43125112 | 39943751 |
|  | (58.21%) | (51.31%) | (50.06%) | (50.41%) | (52.33%) | (52.52%) | (50.17%) | (51.49%) | (50.8%) |
| Splice reads | 22685175 | 32276948 | 29333551 | 31074836 | 30258543 | 27029567 | 27978917 | 27624333 | 26032309 |
|  | (27.11%) | (33.01%) | (34.15%) | (35.61%) | (32.7%) | (31.13%) | (33.29%) | (32.98%) | (33.11%) |
