# Supplementary material for: Identification of candidate genes for milk production traits by RNA sequencing on bovine liver at different lactation stages
Source: BMC Genet. 2020 Jul 9;21:72. doi: 10.1186/s12863-020-00882-y (PMC7346489; doi:10.1186/s12863-020-00882-y)
Supplement: Supplementary file 3 — Additional file 3. Correlation analysis of the reads. [file 12863_2020_882_MOESM3_ESM.docx]

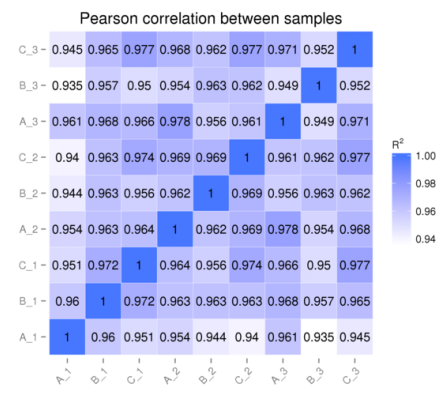


Additional file **3 Correlation analysis of the reads. The** y-axis and the x-axis both correspond to the nine samples. The value at the corner represents the square of Pearson correlation coefficient between the two samples. The higher correlation was represented by the deeper color.
