# Supplementary material for: Identification of candidate genes for milk production traits by RNA sequencing on bovine liver at different lactation stages
Source: BMC Genet. 2020 Jul 9;21:72. doi: 10.1186/s12863-020-00882-y (PMC7346489; doi:10.1186/s12863-020-00882-y)
Supplement: Supplementary file 4 — Additional file 4. Volcano plot displaying differential expressed genes in bovine liver at different lactation stages. [file 12863_2020_882_MOESM4_ESM.docx]

**A**

**early lactation vs. dry period**


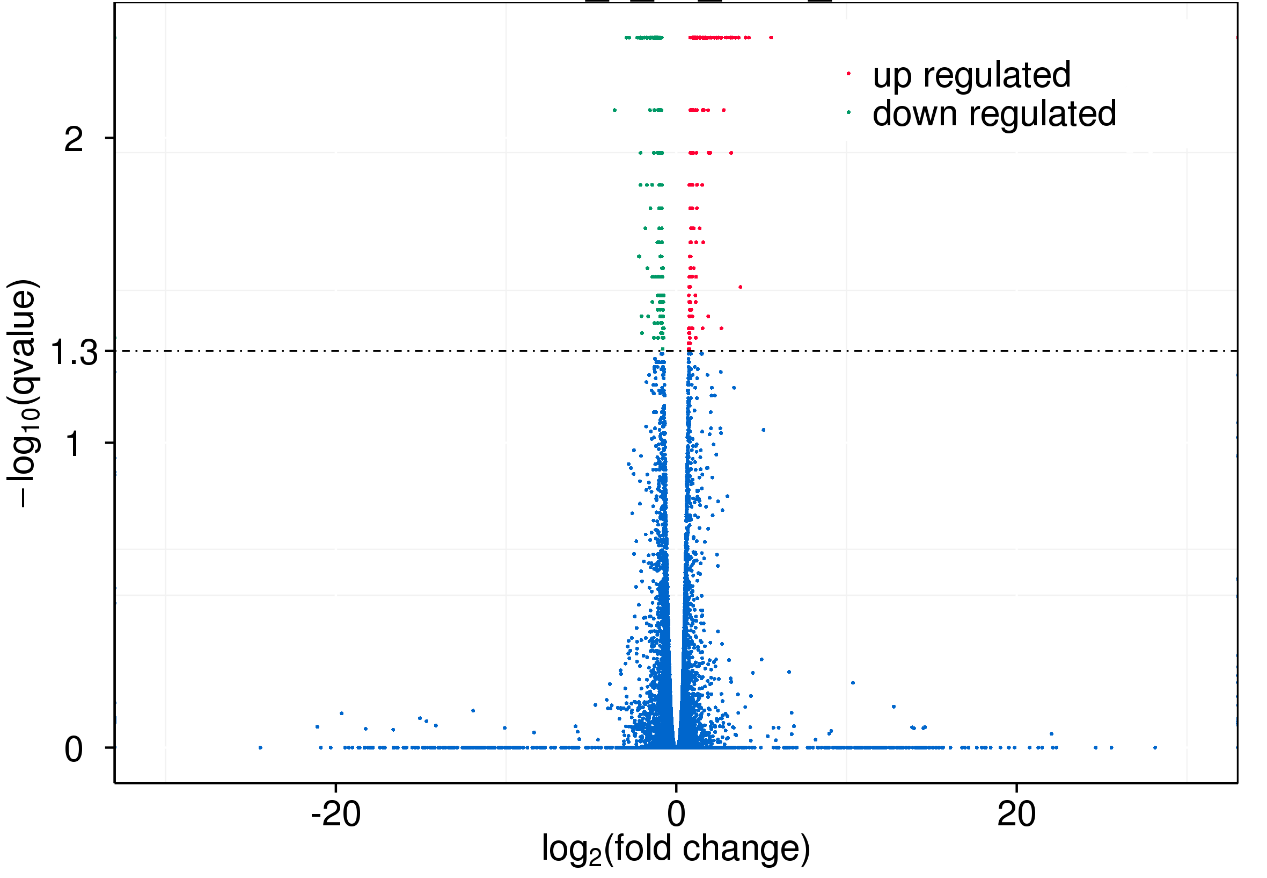


**B**

**peak of lactation vs. dry period**


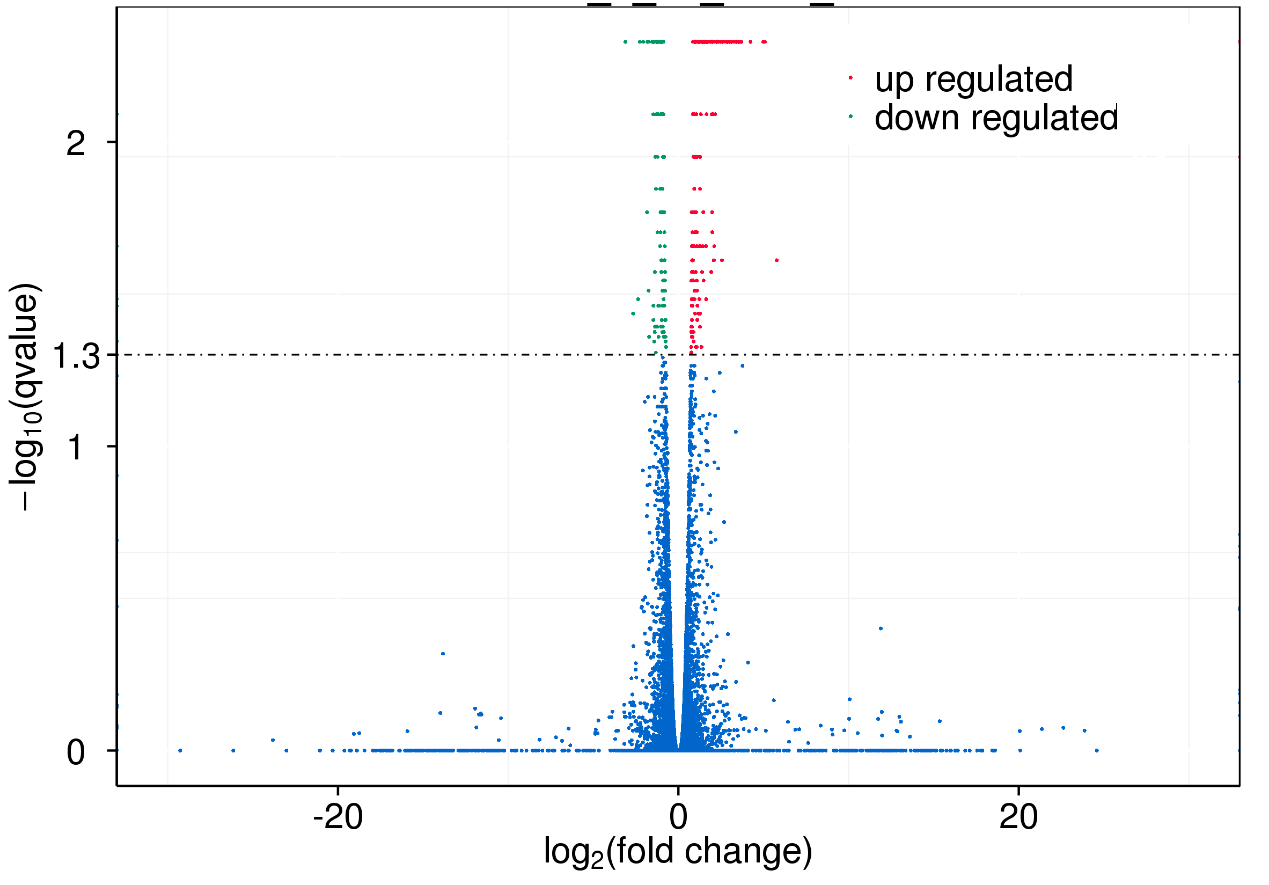


**C**

**peak of lactation vs. early lactation**


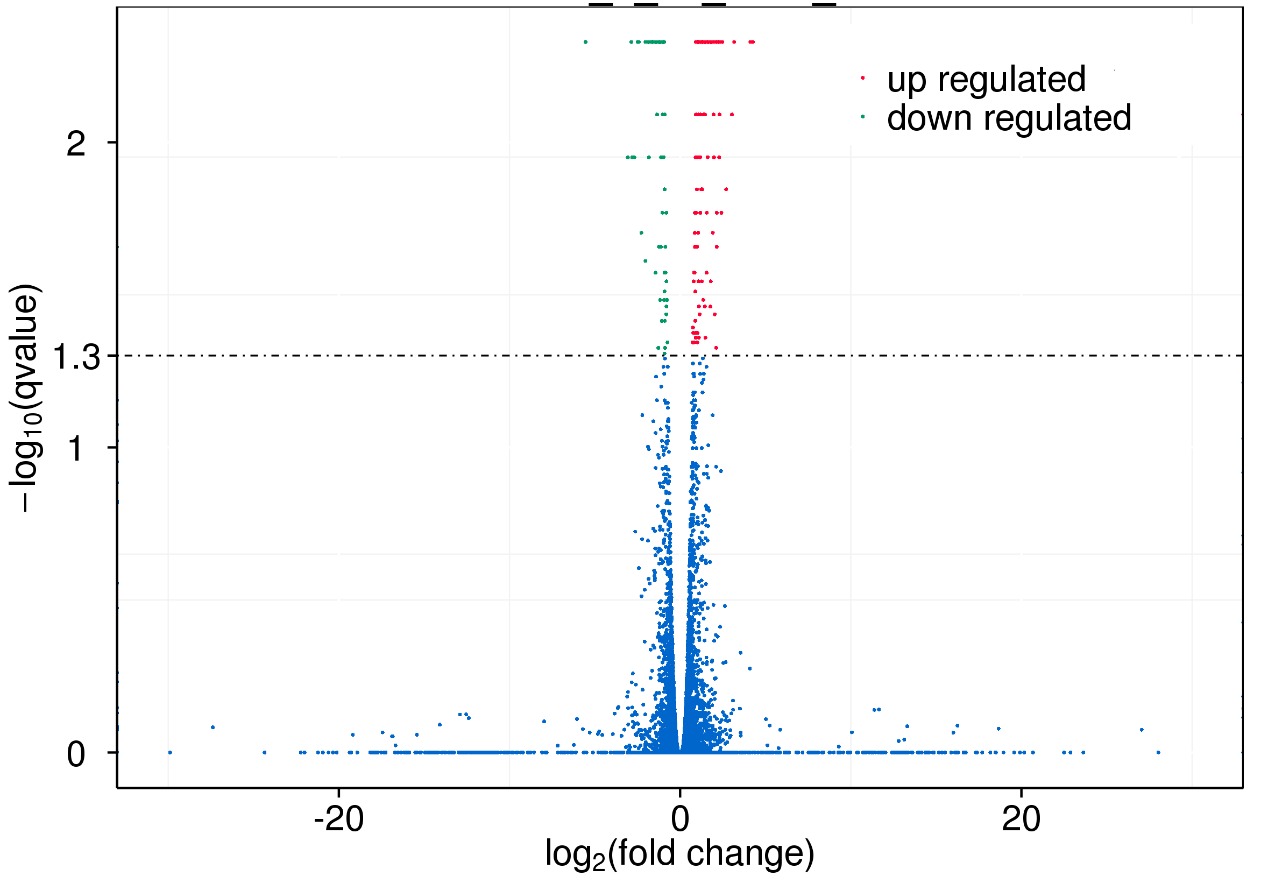


Additional file **4 Volcano plot displaying differential expressed genes in bovine liver at different lactation stages. Red dots represent up regulated genes; green dots represent down regulated genes. (A)** early lactation vs. dry period; (B) peak of lactation vs. dry period ; (C) peak of lactation vs. early lactation.
